# A desmoid-type fibromatosis in the retroperitoneum of the gastro-pancreatic region that was resected with a distal pancreatomy: a case report^☆^

**DOI:** 10.1016/j.radcr.2022.03.066

**Published:** 2022-05-20

**Authors:** Toru Imagami, Takeshi Togawa, Yasumitsu Oe, Akiyoshi Mizumoto, Michiko Hino, Shizuki Takemura

**Affiliations:** a Department of Digestive surgery and Peritoneal dissemination center, Kusatsu General Hospital, 1660 Yabase, Kusatsu City, Shiga 525-8585, Japan; b Department of Pathology, Kusatsu General Hospital, Kusatsu City, Japan

**Keywords:** Desmoid-type fibromatosis, Desmoid tumor, Distal pancreatectomy

## Abstract

An 80-year-old man was referred to our hospital because of epigastric pain. Abdominal computed tomography revealed a well-defined circular intra-abdominal mass in the gastro-pancreatic region measuring 15 mm in diameter. After 6 months, the mass lesion was growing with mild enhancement, and weaker enhancement was found in the lower half of the mass on contrast-enhanced computed tomography. The mass lesion touched the stomach, whereas adipose tissue appeared to intervene between the mass and pancreas. On magnetic resonance imaging, the well-defined mass lesion had isointensity to muscle on T1-weighted imaging, slight hyperintensity to muscle on T2-weighted imaging, which indicated a rich fibrous tumor. Under general anesthesia, the patient underwent open surgery. Intraoperatively, the tumor was separated from the stomach and firmly attached to the pancreas. Therefore, we performed a distal pancreatomy with splenic resection. Pathological diagnosis was desmoid-type fibromatosis in the retroperitoneum, and the tumor margin was attached to the pancreas, splenic artery, and splenic vein. Since there are few reports of desmoid-type fibromatosis occurring in the retroperitoneum of the gastropancreatic region, it is difficult to distinguish from other soft tissue tumors and to identify the tumor origin. Close observation by radiological re-valuation was a useful option. Magnetic resonance imaging signals and an enhanced pattern may help distinguish a desmoid-type fibromatosis from other soft tissue tumors. A desmoid-type fibromatosis that is well-defined in radiological findings may infiltrate the surrounding organs with gross or pathological analyses.

## Introduction

A desmoid-type fibromatosis (DTF) is a tumor-like proliferating fibrous tissue disorder that originates from the connective tissue of the fascia or aponeurosis [1]. A DTF can occur in any of the fibrous connective tissues throughout the body [2]. According to the World Health Organization (WHO), a DTF is characterized by infiltrative growth and a tendency toward local recurrence; however, it is unable to metastasize [3]. Local infiltration of DTF can cause death through invasion of adjuvant vital structures and organs [4]. According to radiology, they are usually non-specific, slow growing solid masses that are difficult to distinguish from other soft tissue tumors [5].

Here, we present a rare case of a slow growing DTF arising from the retroperitoneum in the gastropancreatic region. Radiologically, the lesion did not infiltrate the surrounding organs. However, intraoperative findings revealed a distal pancreatomy that required resection, and the tumor margin was pathologically in contact with the pancreas, splenic artery, and splenic vein, which indicated a dissociation between the preoperative findings and postoperative findings. We report the clinical experience of this case for improvement of radiological diagnoses.

## Case presentation

**Fig. 1:**
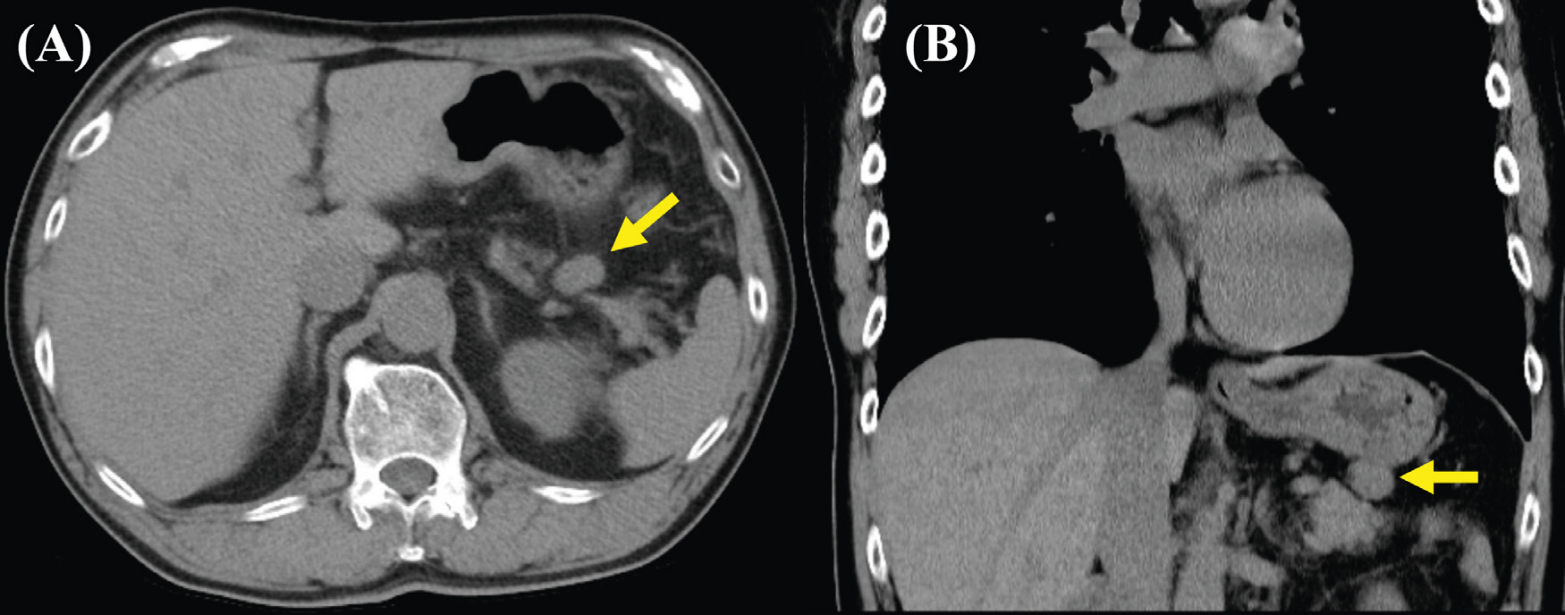
Abdominal CT findings Abdominal axial (A) and coronal (B) CT revealed well-defined round mass in the gastropancreatic region measuring 15 mm in diameter (yellow arow).

**Fig. 2:**
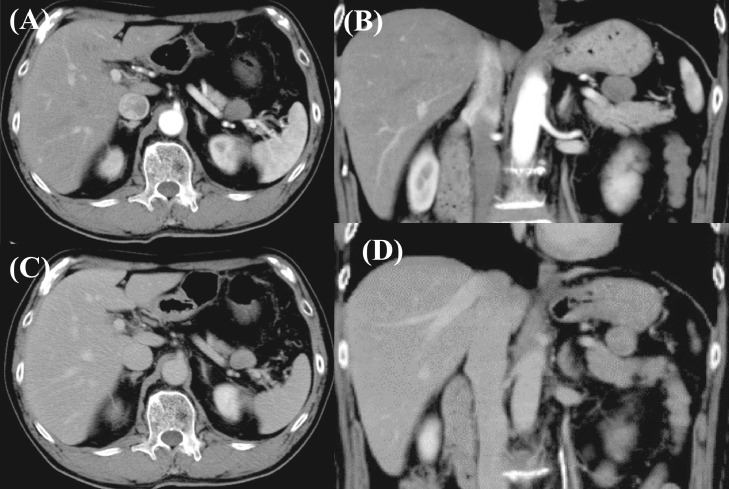
Contrast-enhanced CT findings 4 months after initial CT. The tumor had grown to 19 × 23 × 20 mm in diameter. In early phase, the mass was poorly enhanced on axial sectioning (A) and coronal sectioning (B). In late phase, the mass was enhanced and similar to pancreas on axial sectioning (C) and on coronal sectioning (D).

**Fig. 3:**
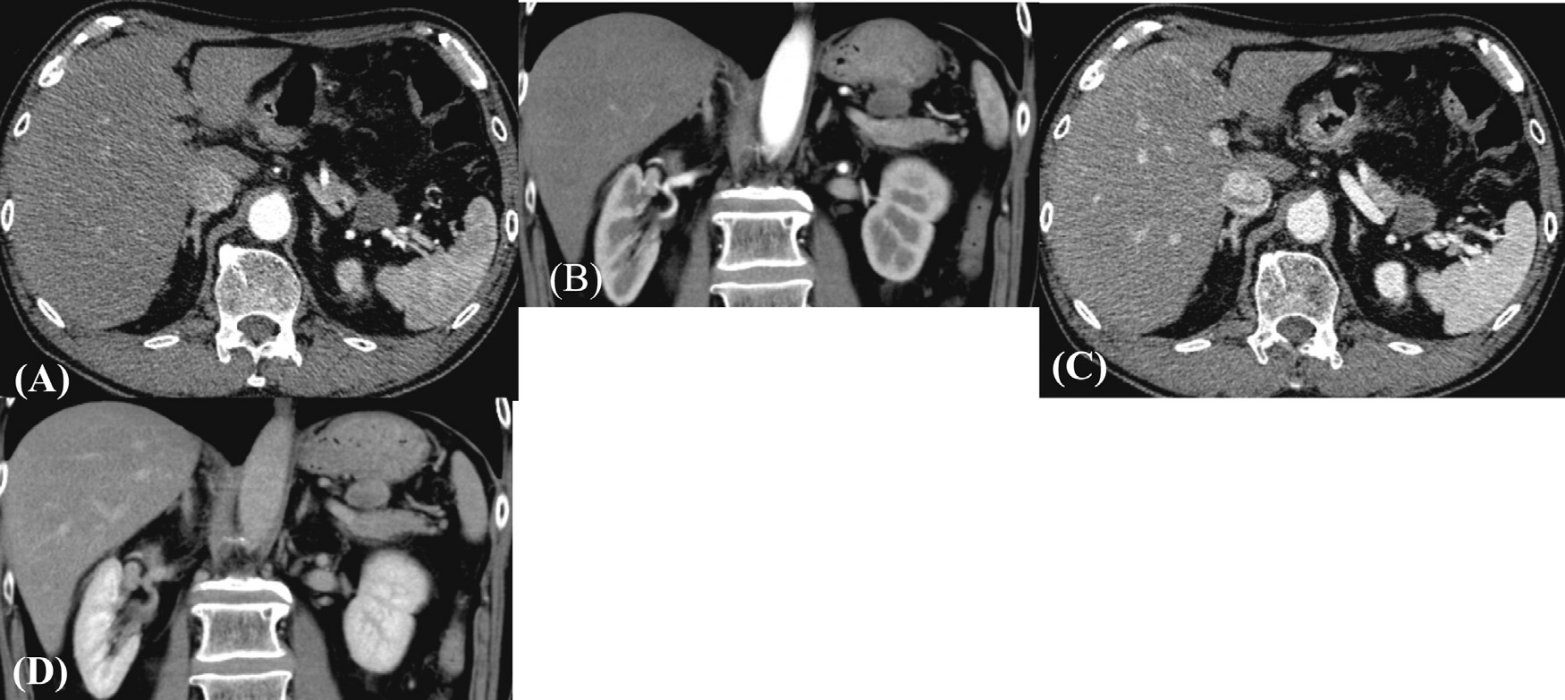
Contrast-enhanced CT findings 6 months after initial CT. Early phase axial section (A) and coronal section (B). Late phase axial section (C) and coronal section (D). The mass suspected to be of retroperitoneum origin and was growing slightly. It gradually enhanced from early phase to late phase with mild enhancement. The enhanced pattern was heterogeneous; namely, the lower half of the mass had weaker enhancement.

**Fig. 4:**
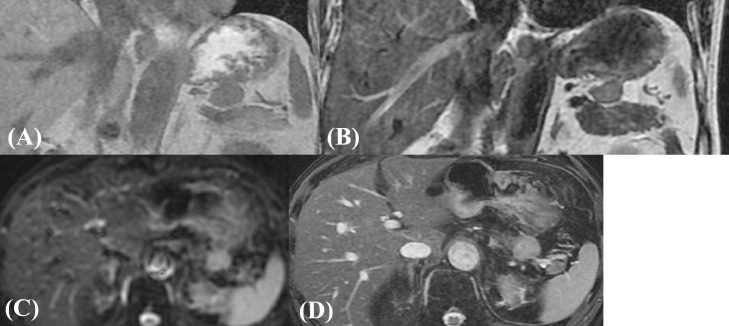
MRI findings (A) T1WI, (B) T2WI, (C) DWI, (D) FIESTA MRI revealed a well-defined mass that had iso-intensity to muscle on T1WI, slight hyper-intensity to muscle on T2WI, and slight hyperintensity on DWI. There was adipose intervening between the tumor and the stomach or pancreas.

**Fig. 5:**
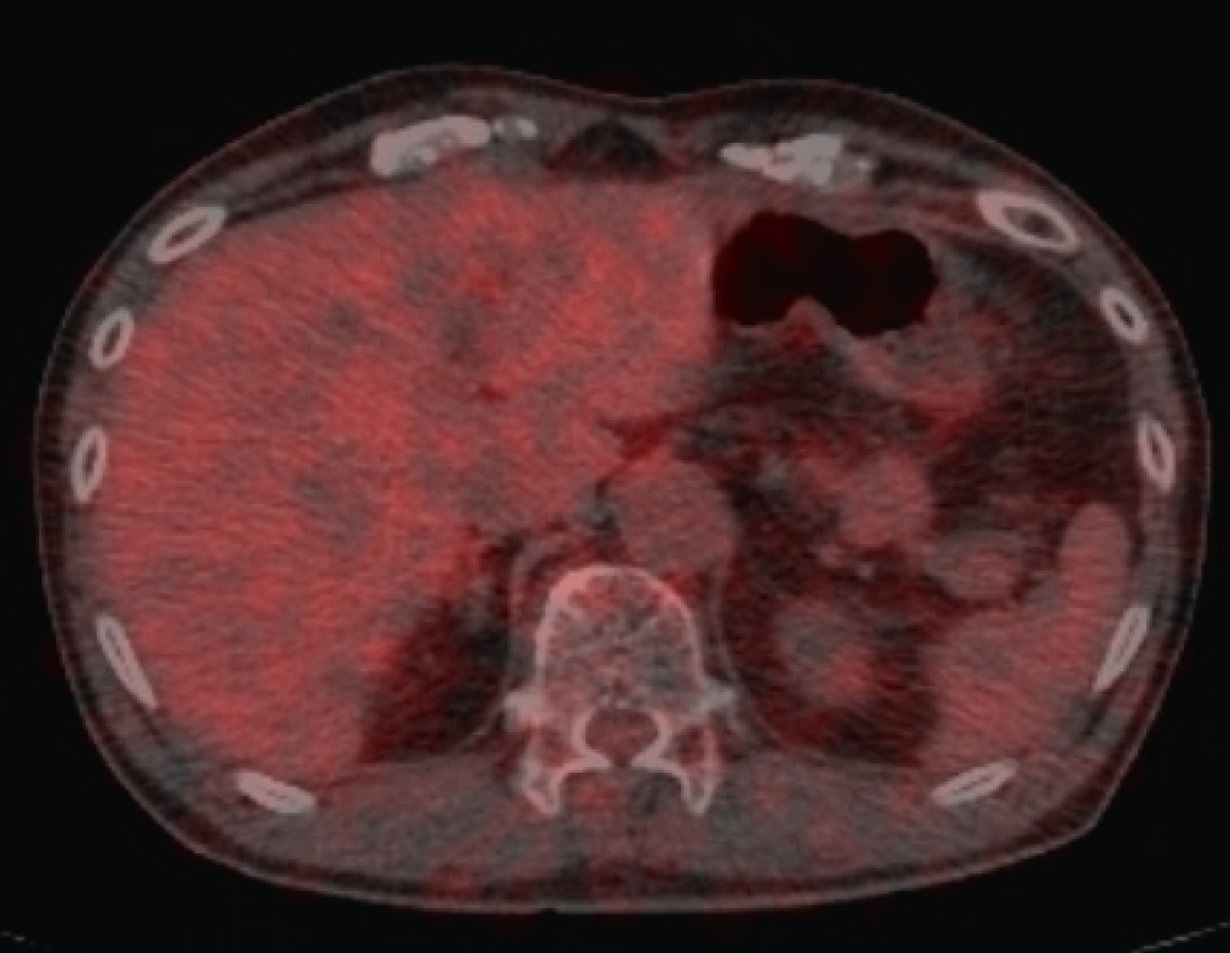
PET/CT showed no abnormal uptake.

An 80-year-old man was referred to our hospital because of epigastric pain. He had a medical history of hypertension and hyperlipidemia. His surgical history included endoscopic mucosal resection for early gastric cancer. Laboratory assessment was within normal limits including tumor markers. Abdominal computed tomography (CT) revealed a well-defined circular intra-abdominal mass in the gastropancreatic region measuring 15 mm in diameter (Fig. 1). After 4 months, abdominal CT showed that the mass lesion had grown to 19 × 23 × 20 mm in diameter. The mass lesion touched the stomach. On the other hand, adipose tissue appeared to intervene between the mass and pancreas. On contrast-enhanced CT, the mass lesion was poorly enhanced in the early phase; however, the mass lesion was enhanced similar to the pancreas in the late phase (Fig. 2). After 2 months, the patient came to our department for surgical treatment. On re-valuation of contrast-enhanced CT, the mass lesion was thought to be of retroperitoneum origin, was growing slightly with mild enhancement, and weaker enhancement was found in the lower half of the mass (Fig. 3). On magnetic resonance imaging (MRI), the well-defined mass lesion had isointensity to muscle on T1-weighted imaging (T1WI), slight hyper-intensity to muscle on T2-weighted imaging (T2WI), and slight hyperintensity on diffusion weighted imaging (DWI; Fig. 4). Positron emission tomography (PET)/CT showed no abnormal uptake (Fig. 5). Based on these radiological findings, the mass lesion was of unknown origin, had a well-defined border, and showed no sign of organ invasion.

We recommended surgical resection to the patient for the following reasons: (1) a definitive diagnosis was difficult radiologically, (2) the tumor grew gradually within 6 months, and (3) the tumor could be resected without organ resection.

**Fig. 6:**
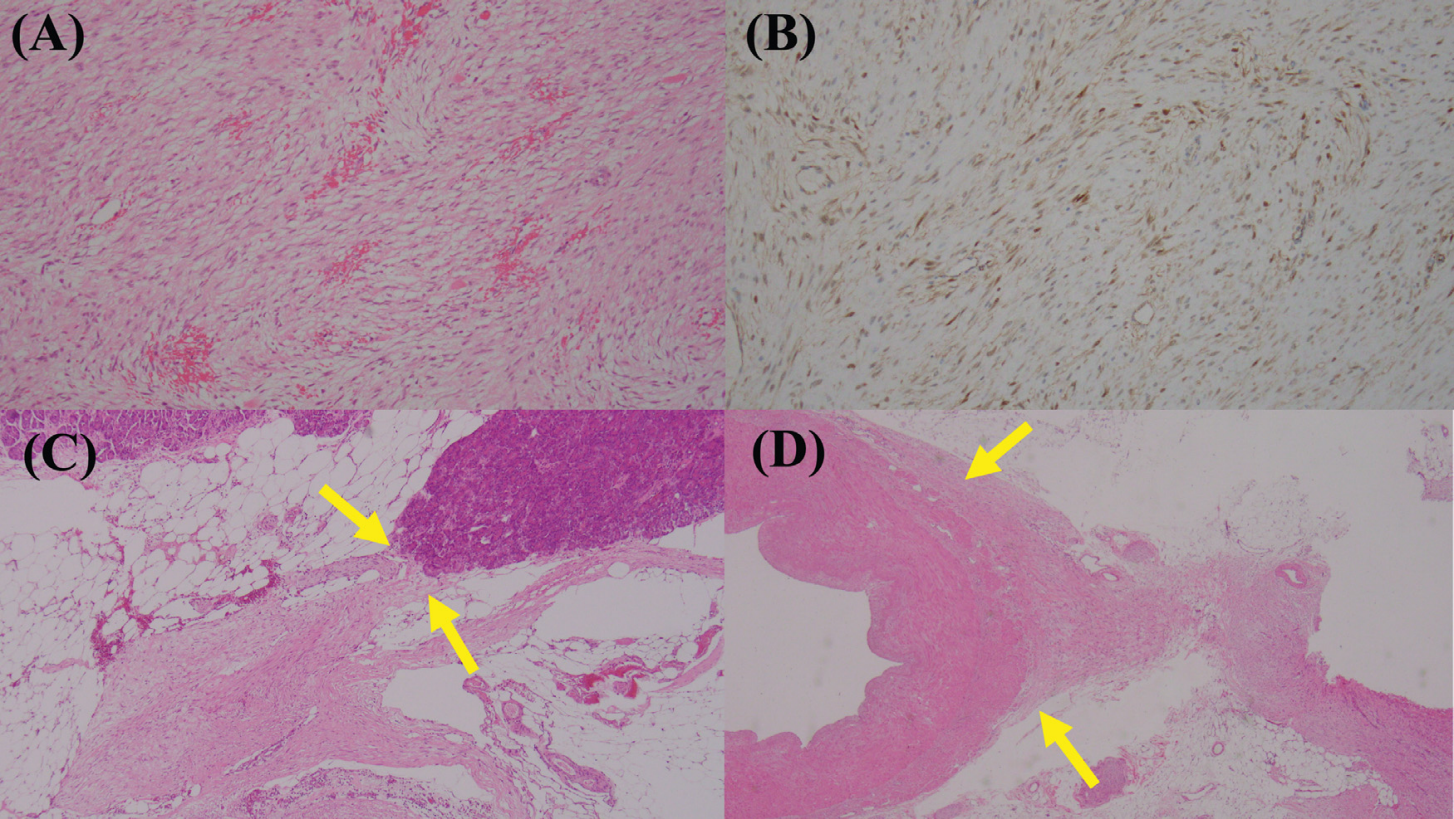
Hematoxylin and eosin stain with 4x revealed abnormal proliferation of spindle-shaped cells (A). Immunohistochemical staining with beta-catenin was positive (B). Desmoid-type fibromatosis attached to pancreas (C, yellow arow) and splenic artery (D, yellow arow).

Under general anesthesia, the patient underwent open surgery. Intraoperatively, the tumor was separated from the stomach and firmly attached to the pancreas. Therefore, we performed a distal pancreatomy with splenic resection. The intraoperative frozen section diagnosis denied a pancreatic invasive ductal adenocarcinoma. On gross section, the tumor had an irregular shape and was 15 × 20 × 35 mm; the cut surface showed a mixture of white and dark red colors. Histological findings showed abnormal proliferation of spindle-shaped cells with a slightly thick fibrous structure without strong atypia (Fig. 6). Microscopically, the tumor margin was attached to the pancreas, splenic artery, and splenic vein. Immunostaining was positive for β-catenin, negative for α-SMA, and slightly positive for Ki67. Based on these findings, the tumor was diagnosed as a DTF originating from the retroperitoneum.

Postoperatively, the patient developed a pancreatic fistula and underwent CT-guided drainage 9 days after surgery. Under local anesthesia in a right lateral position, an 8.5Fr J type catheter was inserted. The pancreatic fistula was treated with drainage and medical therapy. The patient was discharged 48 days after surgery.

## Discussion

A DTF is a locally aggressive fibroblastic proliferation that is characterized by a variable and sometimes unpredictable clinical course [6]. In some instances, the lesions remain stable for long periods with no intervention, while in other cases, the lesions grow rapidly [7]. A DTF is difficult to diagnosis preoperatively due to the lack of typical features; therefore, this case contributed to recognition of the difficulty of a radiological diagnosis.

A DTF is a distinct rare entity that accounts for 0.03% of all tumors and 3% of all soft tissue tumors, with an incidence of 2-4 cases per million per year in the general population [8]. The intra-abdominal type is around 8% of all DTFs [9]. Retroperitoneal DTFs account for less than 1% of retroperitoneal masses [10]. Most DTFs arise sporadically; however, between 5% and 15% are associated with a familial adenomatous polyposis [5]. A history of trauma to the site of the tumor, often surgical in nature, is noted in approximately 25% of the cases [8,11].

Imaging plays an important role in the management of a DTF; the most common imaging modalities are CT and MRI, as well as ultrasonography in select cases [12]. The imaging findings of a DTF depend on the number of fibroblasts proliferating in the tumor, as well as the fiber composition, collagen content, and tumor supply [1]. On CT, a DTF usually appears as a well-circumscribed solid mass with homogeneous density. Contrast-enhanced CT demonstrates typical isoattenuation to hyperattenuation to muscle, commonly demonstrating mild to moderate enhancement [8]. The soft tissue component often appears similar to a solid tumor, such as a gastrointestinal stromal tumor, lymphoma, or soft tissue sarcoma [13]. On MRI, a DTF shows low- to isosignal intensity to muscle on T1WI, and iso- to hypersignal intensity to muscle on T2WI1 [14]. Although there is little data on diffusion weighted MRI, the apparent diffusion coefficients of DTFs were reported to be higher than those of other soft tissue sarcomas [12]. On PET/CT, a DTF is not typically metabolically active and often demonstrates standardized uptake values of less than or equal to 4.8 [12].

Regarding the radiological CT findings in our case, we suspected the mass lesion was a metastatic lymph node, malignant lymphoma, Castleman's disease, or gastrointestinal stromal tumor. The MRI findings indicated a rich fibrous tumor such as Castleman's disease, a solitary fibroma, and a DTF. Among them, the enhanced patterns were not similar to Castleman's disease and a solitary fibroma; however, we could not confirm a diagnosis of DTF. The diagnostic accuracy of “first impression” based on MRI was reported to be higher than that based on CT [15]. This also applies to our case; namely, the high fibrous component in MRI was an important finding. The high fibrous component in MRI signals and the enhanced pattern might have led to a diagnosis of DTF, retrospectively. MRI on DWI and PET/CT was consistent with known findings; however, the findings were considered to be non-specific.

The most common origin of an intra-abdominal DTF is from mesenteric or retroperitoneum connective tissue [9]. There has been a previous report of a DTF that presented in the gastro-pancreatic region. Sugimachi reported a case of DTF originating from the stomach [5]. On the other hand, Mizuno reported a DTF originating from the pancreas [14]. Neither was able to diagnose the origin preoperatively. In our case, the lesion was observed radiologically to be independent of the stomach and pancreas. The lesion was intraoperatively associated with the pancreas, and pathological findings were similar. Although the DTF appeared well-defined at gross analysis, at the microscopic level, its margins appeared to infiltrate the adjacent structures [2,13]. From our experience, DTFs may extend to the surrounding tissues, even if preoperative imaging shows a well-defined margin.

According to the desmoid tumor working group, the treatment options for intra-abdominal or retroperitoneal DTFs are surgery, radiotherapy, and medical therapy [16]. Medical therapy comprises nonsteroidal anti-inflammatory drugs, anti-hormonal therapies, tyrosine kinase inhibitors, and chemotherapeutic regimens [17]. In a previous report, up to 65% of patients with a DTF did not progress, suggesting that some patients do not require any intervention[7]. Therefore, watchful waiting has been suggested as the initial step in recent years. CT is used for monitoring an intra-abdominal DTF [12], and the first clinical and/or radiologic re-valuation should be done within 8-12 weeks, and then every 3 months in the first year [18]. However, delaying treatment might risk the loss of a therapeutic window [19]. Hence, a multidisciplinary approach personalized to the individual patient is required for optimal care [12]. In the case of progression, surgery remains the main treatment for an intra-abdominal DTF [18]. In our case, we could not make a diagnosis pathologically before surgical resection. Instead, of a pathological diagnosis, repeated abdominal CT revealed mass growth at four and 6 months after the initial CT. We suggest that the management with surgical resection should not change even if histopathologic confirmation was obtained after the initial CT. Endoscopic ultrasound-guided fine needle aspiration was not performed due to the risk of vascular injury and tumor dissemination. Radiologic re-valuation seemed to be a useful alternative to biopsy.

The recurrence rate is 15%-30% for an intra-abdominal DTF[13]. In a previous report, R0 resection was associated with longer local recurrence-free survival compared to R1 resection for small tumors (<5 cm), whereas the resection margin was not associated with recurrence in larger lesions [7]. A postoperative nomogram including tumor size and site and patient age was proposed to predict local recurrence [7]. From this nomogram, the recurrence rate of the patient was low and predicted to be about 90% for 3-year and 7-year local recurrence-free survival. Our patient will be followed up in the outpatient department.

This is a single case report, which is a limitation of this study. There are only a few reports of DTF in the retroperitoneum of the gastropancreatic region, making it indistinguishable from other tumors. Thus, accumulation of cases is necessary to improve imaging diagnoses of DTFs in the retroperitoneum of the gastropancreatic region.

## Conclusion

The clinical course of DTFs is variable and unpredictable; therefore, a multidisciplinary approach personalized to the individual patient is required. Close observation by radiological re-valuation was a useful option for a well-defined tumor in the retroperitoneum of the gastropancreatic region, which was pathologically diagnosed as a DTF after surgical resection. MRI signals and an enhanced pattern may help distinguish a DTF from other soft tissue tumors. A DTF that is well-defined in radiological findings may infiltrate the surrounding organs with gross or pathological analyses.

## Patient consent statement

The written informed consent was obtained for publication.

## References

[1] Wang J, Huang Y, Sun Y, Ge Y., Zhang M. (2020). Value of imaging findings in predicting post-operative recurrence of desmoid-type fibromatosis. Oncol Lett.

[2] Hagiwara Katsuhiro, Mihara Kisyo, Aiura Koichi, Shito Masaya (2020). Successful outcomes after laparoscopic spleen-preserving pancreatic resection for a desmoid tumor: a case report. Int J Surg Case Rep.

[3] Jo VY, Fletcher CD (2014). WHO classification of soft tissue tumours: an update based on the 2013 (4th) edition. Pathology.

[4] Meyer A, Szajnbok P, Koszka A J M, Pezzutti D., Segatelli V. (2021). A rare sporadic pancreatic desmoid fibromatosis with splenic vein invasion diagnosed by CT scan-guided core needle biopsy: a case report with possible differential diagnosis from metastatic colorectal or renal cancer. J Surg Case Rep.

[5] Sugimachi K, Iguchi T, Ohta M, Mano Y, Hisano T (2020). Laparoscopic spleen-preserving distal pancreatectomy for a solid-cystic intraabdominal desmoid tumor at a gastro-pancreatic lesion: a case report. BMC Surg.

[6] Khanna K, Abdollahi Mofakham F, Gandhi D (2020). Desmoid fibromatosis of the pancreas––a case report with radiologic-pathologic correlation. Radiol Case Rep.

[7] Crago AM., Denton B, Salas S, Salas A, Mezhir JJ, Hameed M (2013). A prognostic nomogram for prediction of recurrence in desmoid fibromatosis. Ann Surg.

[8] Park CG, Lee YN, Kim WY (2021). Desmoid type fibromatosis of the distal pancreas: a case report. Ann Hepatobiliary Pancreat Surg..

[9] Alghamdi HM. (2021). Invasive giant pancreatic desmoid-type fibromatosis with curative resection: a case report. Int J Surg Case Rep.

[10] Rajiah P, Sinha R, Cuevas C, Dubinsky TJ, Bush WH, Kolokythas O (2011). Imaging of uncommon retroperitoneal masses. Radiographics.

[11] Lopez R, Kemalyan N, Moseley HS, Dennis D, Vetto RM (1990). Problems in diagnosis and management of desmoid tumors. Am J Surg.

[12] Braschi-Amirfarzan M, Keraliya AR, Krajewski KM, Tirumani SH, Shinagare AB, Hornick JL (2016). Role of imaging in management of desmoid-type fibromatosis: a primer for radiologists. Radiographics.

[13] Kyu-Chong L, Jongmee L, Baek Hui K, Ah Kim K, Min Park C (2018). Desmoid-type fibromatosis mimicking cystic retroperitoneal mass: case report and literature review. BMC Med Imaging.

[14] Mizuno M, Kawaguchi Y, Kawanishi A, Kawashima Y., Maruno A., Ogawa M. (2017). Intra-abdominal desmoid tumor, embedded in the pancreas, preoperatively diagnosed as an extragastric growing gastrointestinal stromal tumor. Case Rep Oncol.

[15] Xu H, Jung Koo H, Lim S, Wook Lee J, Na Lee H, Kwan Kim D. (2015). Desmoid-type fibromatosis of the thorax: CT, MRI, and FDG PET characteristics in a large series from a tertiary referral center. Medicine (Baltimore).

[16] Desmoid Tumor Working Group (2020). The management of desmoid tumours: a joint global consensus-based guideline approach for adult and paediatric patients. Eur J Cancer.

[17] Shen C, Wang C, Yan J, He T, Zhou X, Ma W (2019). Clinicopathological characteristics, treatment, and survival outcomes of retroperitoneal desmoid-type fibromatosis: a single-institution experience in China. Medicine (Baltimore).

[18] Kasper B, Baumgarten C, Garcia J, Bonvalot S, Haas R, Haller F (2017). An update on the management of sporadic desmoid-type fibromatosis: a European Consensus Initiative between Sarcoma PAtients EuroNet (SPAEN) and European Organization for Research and Treatment of Cancer (EORTC)/Soft Tissue and Bone Sarcoma Group (STBSG). Ann Oncol.

[19] Grignol VP, Pollock R, Howard JH (2016). Management of desmoids. Surg Clin North Am..

